# The diversity of vaginal microbiome in women infected with single HPV and multiple genotype HPV infections in China

**DOI:** 10.3389/fcimb.2022.642074

**Published:** 2022-12-19

**Authors:** Shufa Liu, Yuanyue Li, Yuzhu Song, Xiaomei Wu, Zulqarnain Baloch, Xueshan Xia

**Affiliations:** ^1^ Faculty of Life Science and Technology and Yunnan Provincial Center for Molecular Medicine, Kunming University of Science and Technology, Kunming, Yunnan, China; ^2^ Department of Gynecology, The First People’s Hospital of Yunnan Province, Kunming, Yunnan, China; ^3^ Yunnan Provincial Key Laboratory of Clinical Virology, Kunming, Yunnan, China

**Keywords:** vaginal microbiota, human papillomavirus (HPV), single infection, multiple infections, Cervical Cancer

## Abstract

**Introduction:**

The human papillomavirus (HPV) is the leading cause of cervical cancer globally. However, its microbial composition and association with the types of HPV infection remain elusive.

**Methods:**

This study was designed to characterize the vaginal microbiota of 53 HPV-infected and 16 normal women (control group) by using high-throughput sequencing with the Illumina platform.

**Results:**

In this study, the five leading phyla were *Firmicutes* (73.9%), *Actinobacteriota* (12.8%), *Proteobacteria* (6.2%), *Fusobacteria* (3.5%), and *Bacteroidota* (3.1%). We found that single HPV genotype–positive women had higher α-microbial diversity compared with HPV-negative and multiple HPV–positive women. In women with a single HPV genotype infection, the HPV-16 infection had significantly higher α-diversity than other genotype infections. In multiple HPV genotype–positive women, the highest α-diversity was found in women positive for HR–HR HPV genotype infection, compared with other infections. Furthermore, in single- and multiple-genotype infections, the abundance of s_unclassified_g_Lactobacillus decreased whereas the abundance of s_Gardnerella_vaginalis increased compared with control. Additionally, s_unclassified_f_Rhizobiaceae and s_sneathia_sanguinegens were only found in HPV-infected women.

**Conclusion:**

This study showed that the type of HPV infection was associated with the composition of the vaginal microbiota. Further studies on HPV genotypes and vaginal microbiota are necessary to uncover more mysteries of their association and provide a promising therapeutic target as well as low-cost future therapeutic strategies.

## Background

Cervical cancer is the fourth most common female cancer in the world. According to estimates, 570,000 cervical cancer cases and 311,000 deaths were reported in 2018 (Arbyn et al., 2020). Human papillomavirus (HPV) most commonly causes cervical squamous cell carcinoma (SCC) and its precursor lesions (cervical intraepithelial neoplasia; CIN) worldwide (zur Hausen, 2002; Muñoz et al., 2003; Bruni L et al., 2010; Forman et al., 2012; Crosbie et al., 2013). HPV, a small, double-stranded, non-enveloped DNA virus, infects genital tracts and oral mucosa. HPV genotypes have been divided into two groups depending on their carcinogenicity, i.e., high-risk and low-risk genotypes (Bodily and Laimins, 2011). The most frequent genotype is HPV-16 globally (Crow, 2012); however, the prevalence of other HPV genotypes varies from region to region (Crow, 2012; Baloch et al., 2016). According to estimates, most HPV infections are transient and cleared within a couple of years (Crow, 2012) (Myers et al., 2000; Richardson et al., 2003). Only 10%–20% of HPV infections persist latently, leading to disease progression and invasive cancer development (Shanmugasundaram and You, 2017). However, the underlying molecular mechanisms of HPV persistent infection are still not well understood.

The vaginal tract is populated with various types of microorganisms, which are collectively called the vaginal microbiome. It has been reported that vaginal microbiota protects women from different urogenital infectious diseases (Watts et al., 2005; Gillet et al., 2011; Guo et al., 2012; Lee et al., 2013; Vriend et al., 2015). The role of vaginal microbiota in the acquisition and persistence of HPV has been reported (Brotman et al., 2014; Mitra et al., 2016). However, the role of the diverse vaginal microbiome in single HPV and multiple HPV genotype infection has not been adequately investigated. Therefore, the current study was designed to investigate whether single-HPV and multiple HPV–genotype infections are associated with the diverse community of vaginal microbiota composition.

## Methods

### Ethical statement

This study was approved by the Ethics Committee of the Faculty of Life Science and Technology, Kunming University of Science and Technology, and the Center for Disease Control and Prevention (CDC) in Yunnan Province, China. Written consent was individually obtained from all participants.

### Study design

A total of 69 women were recruited who visited the Out-Patient Department of the First People’s Hospital of Yunnan Province from November 2016 to July 2017. For the sample collection, the vaginal swab was inserted ~2–3 cm into the vagina and swirled for ~30 s. The swab samples were immediately put into Fluidx tubes containing 0.8 ml of DNA/RNA Shield and transferred to the laboratory within 30 min. Samples were pelleted by centrifugation at ≥10,000× g (25°C) for 10 min and the sediment stored at −80°C until further analysis. Women infected with HIV, hepatitis B/C, or autoimmune disorders; who received antibiotics or pessaries within 14 days of sampling; or had a previous history of cervical therapy were excluded. A standardized questionnaire was used to collect information from each participant about their ethnicity, education, age, marital status, smoking, drinking, illness history, sexual activity, and profession.

### DNA extraction

Bacterial and viral genomic DNA was extracted from the samples synchronously by using the TIANamp DNA Extraction Kit (Tiangen Biotech Co., Lai Chi Kok, Hong Kong) following the manufacturer’s instructions. Quality control was carried out by gel electrophoresis, and nanograms per microliter of DNA and 260/280 OD were measured using a NanoDrop 1000 spectrophotometer (Thermo Fisher Scientific Inc., US). The purified DNA was stored at −20°C until further HPV genotyping and microbiota sequencing.

### HPV genotype examination

DNA was amplified with broad-spectrum consensus primers (MY09/11) targeting the HPV L1 region using polymerase chain reaction (PCR) (Baloch et al., 2016). Isolated and amplified DNA from HeLa and CaSki cell lines was used as positive controls, and solutions without sample DNA were used as negative controls. GenoArray Test Kit (Hybribio, Chaozhou, China), an L1 consensus primer-based PCR assay kit, was used to amplify 23 HPV genotypes, namely, 13 HR-HPV genotypes (HPV-16, -18, -31, -33, -35, -39, -45, -51, -52, -56, -58, -59, and -68), three PHR-HPV genotypes (HPV-53, -66, and -81) and seven low-risk HPV (LR-HPV) genotypes (HPV-6, 11, 42, 43, 44, and 61), according to the manufacturer’s recommendations.

### 16S rRNA gene sequencing and analysis

In this study, the V4 segment of the 16S rRNA gene was amplified, and the sequencing was performed using the Illumina MiSeq system, according to the manufacturer’s recommendations. FASTQ file conversion of the raw data was performed following demultiplexing using the HiSeq reporter. High-quality reads (80% bases have Q score >20) were selected for analysis, and reads with unknown bases (N) and poor-quality reads were discarded. Sequences were grouped into operational taxonomic units (OTUs) at a similarity threshold of 97%. The SILVA rRNA gene database was used to make taxonomic assignments for all OTUs and phylum, class, order, family, and genus and were identified at the species level.

Based on OTU information, rarefaction curves and α-diversity indices refer to community diversity (Shannon, Simpson), community richness (Chao1 and Ace), community evenness (Shannon-even, Simpson-even), and sequencing depth (Good’s coverage) calculated by Mothur. A heatmap diagram showing the relative abundance of OTUs was generated using the Vegan Package in R 2.4. Phylogenetic beta diversities, including principal coordinate analysis (PCoA), were evaluated with the Bray–Curtis distance using QIIME 1.7.0. The linear discriminant analysis (LDA) and effect size (LEfSe) methods were used for potential biomarker detection. The threshold score of LDA was set at 3.0, and a significant p-value of 0.05 was employed.

### Statistical analysis

A chi-square test was used to assess the differences between demographic and clinical characteristics of participants. Differences in microbiota between groups were measured using PERMANOVA (weighted UniFrac distance). Differences in the Chao index and Shannon index (α-diversity metrics) were tested according to the type of HPV infection using the Wilcoxon rank-sum test. Metagenomic potential biomarker discovery and associated statistical significance were assessed by analyzing the relative taxonomic abundances according to the linear discriminant analysis (LDA) effect size (LEfSe) methods. In LEfSe, the Kruskal–Wallis rank-sum test was used to distinguish features with significantly different taxon abundances in groups and LDA to calculate the size effect of each feature. A threshold of 3.0 on the logarithmic LDA score was used for discriminative microbial biomarkers. The association between microbial community structure and single-HPV-genotype or multiple-HPV-genotype infections was analyzed by multivariable logistic regression. The relationships among HPV infection type, ethnicity, married status, age, smoking, drinking, and sexual partner were compared by Fisher’s exact test.

## Results

### Characteristics of participants

A total of 69 women were enrolled in this cohort study and were divided into three groups: healthy control (n = 16; 23.2%), single HPV-genotype infection (n = 31; 44.9%), and multiple HPV-genotype infection (n = 22; 31.9%). Among the total, single-infection, and multiple-HR-HPV genotypes, infected women were 26 and seven. HR-PHR-HPV-infected women were six, HR-LR-HPV infection was found in nine women, and LR-HPV/PHR-HPV infection was found in five women. Overall, 15, 12, 10, and eight participants were infected with HPV-16, 52, 33, and 58, respectively (**Table 1**). The demographic characteristics of participants are shown in **Table 2**. Among 69 participants, 53 were Han, 16 were from other ethnicities, 48 were married, and 21 were unmarried. Fifty-six women reported having a single sexual partner.

**Table 1:** Different HPV distribution among single infection and multiple infections.

Genotypes	Single infection (n=31)	Multiple infections (n=22)	Total
**HR-HPV**	26	7	33
**HR-PHR-HPV**	3	3	6
**HR-LR-HPV**	0	9	9
**LR-HPV or PHR-HPV**	2	3	5
**HPV-6**	0	1	1
**HPV-11**	0	1	1
**HPV-16**	7	8	15
**HPV-18**	0	2	2
**HPV-31**	1		1
**HPV-33**	6	4	10
**HPV-34**	0	1	2
**HPV-35**	1	2	3
**HPV-43**	0	1	2
**HPV-51**	3	2	5
**HPV-52**	5	7	12
**HPV-53**	0	4	4
**HPV-55**	0	1	1
**HPV-58**	6	2	8
**HPV-59**	0	3	3
**HPV-61**	0	2	2
**HPV-66**	0	3	3
**HPV-68**	1	2	2
**HPV-72**	0	2	2
**HPV-81**	0	3	3
**HPV-82**	1		1
**HPV-83**	0	1	1
**HPV-84**	0	1	1

**Table 2:** Demographic characteristics of participants.

Parameter	Control (n = 16)	Single infection (n = 31)	Multiple infections (n = 22)
Age mean ± SD	35.75 ± 7.94	39.55 ± 10.05	37.82 ± 9.21
Age range	23~49	22~59	23~55
**Ethnicity**			
Han	11 (68.8)	26 (83.9)	16 (72.7)
Others	5 (31.2)	5 (16.1)	6 (27.3)
**Education level**			
Primary or less	4 (25)	9 (29)	4 (18.2)
Middle	7 (43.7)	13 (41.9)	11 (50.0)
College or above	5 (31.2)	9 (29)	7 (31.8)
**Marriage**			
Married	16 (100)	21 (67.7)	11 (50)
Single	0	10 (32.2)	11 (50)
**Smoking**			
Yes	1 (6.2)	1 (3.2)	0 (0.0)
No	15 (93.8)	30 (96.8)	16 (100)
**Drinking**			
Yes	1 (6.2)	3 (9.7)	0 (0.00)
No	15 (93.8)	28 (90.3)	16 (100)
**Sexual partner**			
1	15 (93.8)	23 (74.2)	18 (81.8)
2 or more	1 (6.2)	8 (25.8)	4 (18.2)

### Composition of cervical microbiota

**Figure 1:**
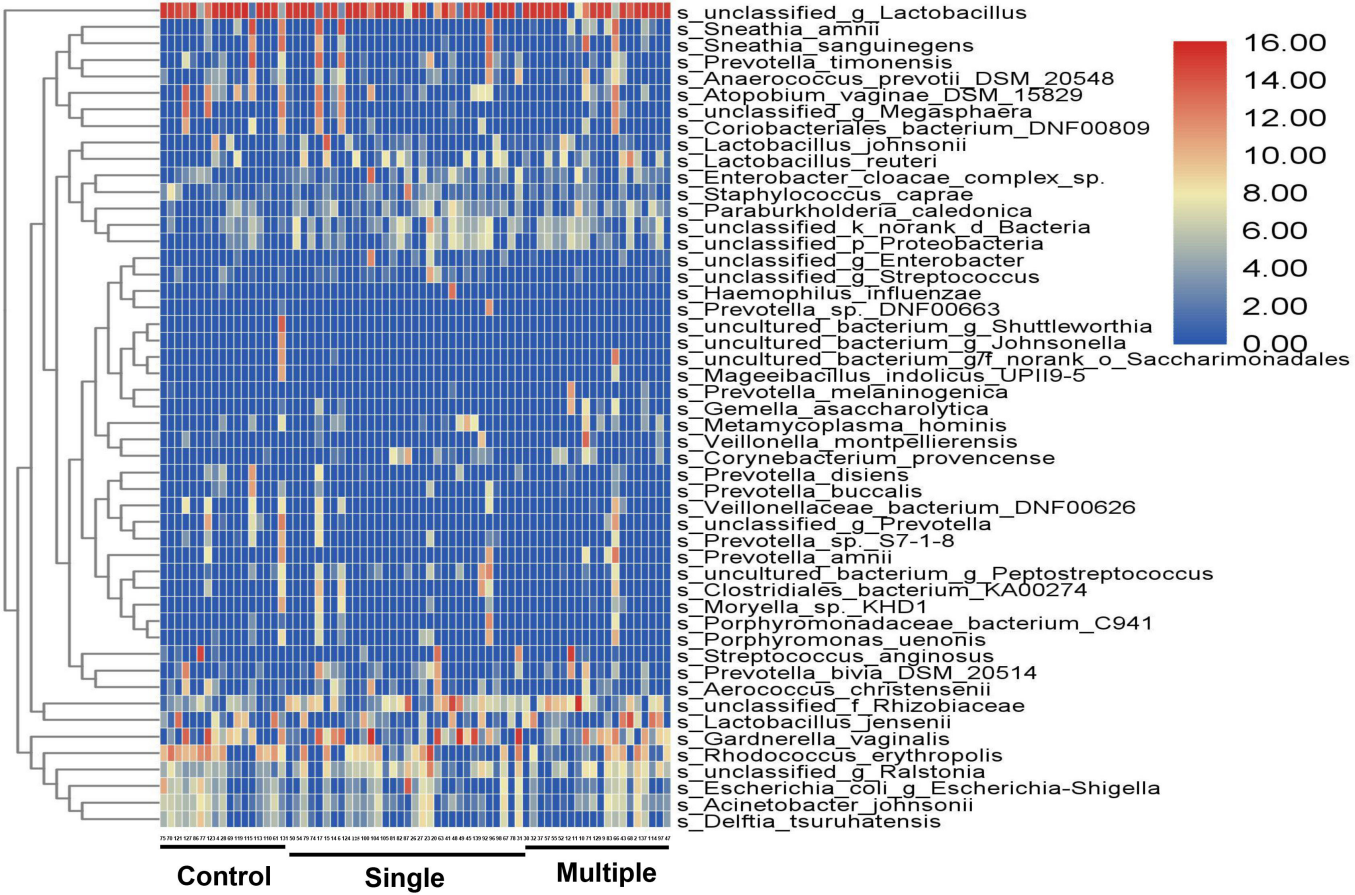
Heatmap shows diverse microbial composition in HPV-infected and control women. Color represents the relative abundance of bacterial specie; red indicates a high proportion, and blue indicates a low abundance.

**Figure 2:**
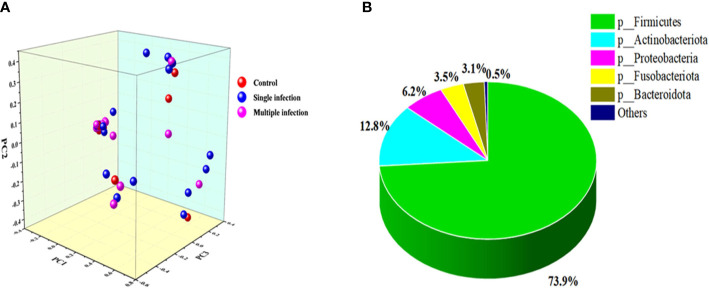
**(A)** PCoA profile of the cohort associated with HPV-infected status displayed with weighted UniFrac distance. Each dot presents a sample, and the color is according to grouping. **(B)** The dominant phyla in the vaginal microbial community.

A 16S rRNA targeted metagenomics analysis was performed on individual vaginal swab samples collected from 69 women and allowed a comparison of the overall bacterial richness and phylogenetic composition of the vaginal microbiota. A heatmap analysis shows the vaginal microbiota composition, which is different among HPV-negative, single-genotype, and multiple-genotype infections in women (**Figure 1**). For conforming, α-diversity (the index of Sob, Chao, ACE, Shannon, Simpson, Coverage, Shannon, and Simpson even) and β-diversity (PCoA) were analyzed. α-Diversity analysis of the microbiota profile based on Shannon and Chao 1 diversity indicated substantial differences among different groups (**Tables 3**, **4**). For β-diversity analysis, we performed a principal coordinate analysis (PCoA) to confirm the differences. We found a different clustering based on the HPV infection status (**Figure 2A**).

**Table 3:** The α-diversity of the vaginal microbial community compound at the genus level.

Estimators	Control	Single infection	Multiple infections	p-value
Sobs	33.63 ± 14.91	52.13 ± 35.18	47.91 ± 31.57	0.159
Shannon	0.54 ± 0.55	0.70 ± 0.71	0.48 ± 0.64	0.423
Simpson	0.77 ± 0.26	0.71 ± 0.29	0.82 ± 0.24	0.347
Ace	42.59 ± 26.60	74.09 ± 46.68	74.66 ± 58.07	0.069
Chao	39.74 ± 18.95	68.02 ± 41.42	62.54 ± 43.96	0.06
Coverage	0.9999 ± 0.00013	0.9996 ± 0.00024	0.9997 ± 0.0030	0.011
Shannon-even	0.16 ± 0.16	0.17 ± 0.16	0.12 ± 0.14	0.545
Simpson-even	0.06 ± 0.04	0.04 ± 0.02	0.05 ± 0.04	0.38

**Table 4:** The α-diversity of the vaginal microbial community compound at the species level.

Estimators	Control	Single infection	Multiple infections	p-value
Sobs	40.25 ± 20.03	64.16 ± 44.84	58.59 ± 41.26	0.146
Shannon	0.67 ± 0.58	0.80 ± 0.79	0.62 ± 0.64	0.621
Simpson	0.71 ± 0.26	0.69 ± 0.30	0.76 ± 0.25	0.65
Ace	52.39 ± 28.45	94.51 ± 59.97	95.70 ± 93.84	0.05
Chao	47.63 ± 24.08	84.97 ± 51.15	80.68 ± 57.48	0.042
Coverage	0.9998 ± 0.00016	0.9995 ± 0.00030	0.9996 ± 0.00041	0.010
Shannon-even	0.18 ± 0.16	0.19 ± 0.17	0.15 ± 0.15	0.732
Simpson-even	0.05 ± 0.04	0.04 ± 0.03	0.05 ± 0.04	0.414

**Figure 3:**
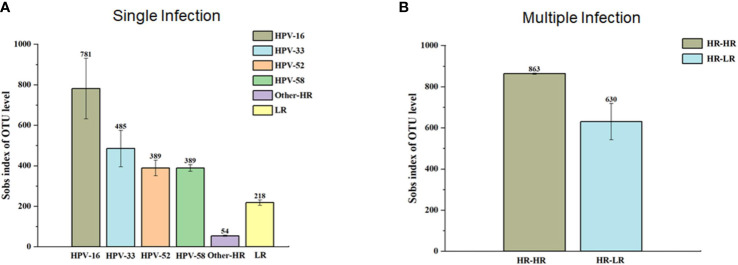
The α-diversity (Sobs index of OUT level) between single infection group **(A)** and multiple infection **(B)** groups. In single infection group, samples with HPV-16 infection presented the highest α-diversity in contrast to HPV-33, 52, 58 and others. In multiple infection groups, the highest α-diversity is in HR-HR HPV infection group compared to HR-HPVE and LR-HPV group.

In this study, a total of 27 phyla, 62 classes, 156 orders, 262 families, 497 genera, 719 species, and 907 OTUs were classified. Among them, the five leading phyla were *Firmicutes* (73.9%), *Actinobacteriota* (12.8%), *Proteobacteria* (6.2%), *Fusobacteria* (3.5%), and *Bacteroidota* (3.1%) (**Figure 2B**). Furthermore, we found that HPV-negative and multiple-genotype-infected women had less microbiota diversity compared with single HPV genotype–infected women (**Figures 3A, B**).

**Figure 4:**
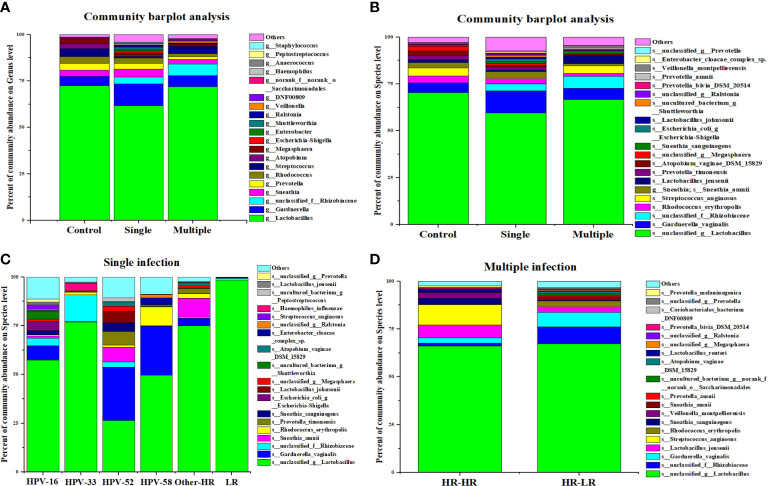
The composition of vaginal microbiota dependent on the HPV infection status. **(A)** Community barplot analysis on genus level among control, single infection and multiple infections. **(B)** Community barplot analysis on species level among control, single infection and multiple infections. **(C)** Community barplot analysis on species level related to HPV genotype in single infection group. **(D)** Community barplot analysis on species level related to genotype combination in multiple infection groups.

At the genus level, we found that *Lactobacillus* and *Gardnerella* were the two leading bacterial genera in control, single HPV infection, and multiple HPV infection groups (**Figure 4A**). However, the abundance of s_unclassified_g_Lactobacillus decreased and that of g_Gardnerella_vaginalis increased in single- and multiple-genotype infections compared with control (**Figure 4B**). Further, g_unclassified_f_Rhizobiaceae was only found in HPV-positive women. Its abundance was higher in multiple HPV genotype–infected women compared with single HPV genotype–infected women (**Figure 4A**).

We further compared vaginal microbial diversity among different HPV genotypes. Our analysis showed that women infected with HPV-16, -52, and -58 had higher microbiota diversity than HPV-33 and other genotypes of infection (**Figure 4C**). Additionally, the proportion of s_Streptococcus_anginosis, s_lactobacillus_jemsenii, s_sneathia_amnii, etc., was higher in multiple HR–HR HPV genotype–infected women compared with LR–LR HPV infection (**Figure 4D**).

### Identification of vaginal microbiota: Potential markers of HPV infection

**Figure 5:**
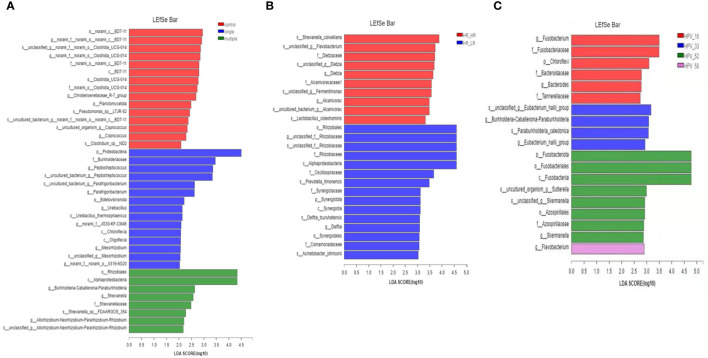
Taxonomic biomarkers. **(A)** The linear discriminant analysis (LDA) effect size (LEfSe) analysis among control, single-infection, and multiple-infection groups. **(B)** The linear discriminant analysis (LDA) effect size (LEfSe) analysis between HR-HPV, HR-HPV and LR-HPV, and HR-HPV genotype infection groups. **(C)** The linear discriminant analysis (LDA) effect size (LEfSe) analysis in single infection associated with the HPV genotype.

In this study, we further extended our investigation to explore the possible bacterial biomarkers for HPV infection; therefore, we compared the relative abundance of different bacteria among women in the single HPV genotype, multiple HPV genotype, and control groups. Linear discriminant analysis (LDA) effect size (LEfSe) modeling was adopted, and 38 clades were detected (**Figure 5A**). We found that p_Proteobacteria, f_Burkholderiaceae, g_Peptostreptococcus, and *s_uncultureed_bacterium_g_Peptostreptococuss* were abundant in single infections, whereas o*_Rhizobiales* and *c_α-Proteobacteria* were more prevalent in multiple HPV genotype infections. Among multiple HR-HPV genotype infections, 10 clades were detected, and 15 clades were identified in multiple HR-HPV and LR-HPV genotype infections (**Figure 5B**). Among single HPV genotype infections, a total of 19 clades were confirmed (**Figure 5C**). These differentially abundant taxa could be considered as potential biomarkers.

## Discussion

The effect of multiple HPV genotype infections on cervical cancer progression is still unclear (Pista et al., 2011). However, according to estimates, multiple HPV genotype infections have been commonly reported among young compared with old women as well as women with an impaired immune system or cytological anomalies (Ho et al., 1998; Herrero et al., 2000; Chaturvedi et al., 2005). The relationship between multiple HPV genotype infections and cervical cancer progression is inconsistent. Some reports have shown that multiple HPV genotypes increase the development of cervical cancer lesions (Fife et al., 2001; van der Graaf et al., 2002; Pista et al., 2011). However, some reports suggested no difference in cervical cancer lesion developments with multiple HPV genotype infection compared with single HPV genotype infection (Herrero et al., 2000; Bosch et al., 2002; Cuschieri et al., 2004; Levi et al., 2004).

In this study, we evaluated the vaginal microbial profiles of single HPV genotype– and multiple HPV genotype–infected subjects for the first time. We applied high-throughput sequencing to evaluate whether single HPV genotype and multiple HPV genotype infections were associated with the diversity of the vaginal microbiota composition. We found that vaginal microbiota composition differed from single HPV genotype and multiple HPV genotype infections compared with controls. Overall, *Lactobacilli* was the most populated species among all participants, but its abundance was decreased in single HPV genotype and multiple HPV genotype infections compared with control, which is in line with previous studies (Lee et al., 2013; Mitra et al., 2015). The Simpson index of α-diversity showed an increasing trend among single HPV genotype and multiple HPV genotype infections and controls. In single HPV genotype–infected subjects, HPV-16-infected subjects showed the highest α-diversity compared with other HPV genotypes. In multiple HPV genotype–infected groups, the highest α-diversity was found in HR–HR HPV–infected subjects. In this study, we further analyzed β-diversity to evaluate species complexity among single HPV genotype–infected, multiple HPV genotype–infected, and control individuals. We observed that the proportion of *Lactobacilli* was decreasing in single HPV genotype– and multiple HPV genotype–infected subjects compared with controls.

With the application of deep metagenomic sequencing, 396 HPV genotypes have been identified (Bzhalava et al., 2014). Among them, 14 HPV genotypes are considered high-risk due to their oncogenic potential. In this study, HPV-16 was the most prevalent genotype, in line with previous research (Crow, 2012). Generally, microbial diversity is considered a sign of health (Turnbaugh et al., 2007). However, the female reproductive system was dependent on the lower diversity of the microbial community and was dominated by specific microorganisms (Mendling, 2016; Miller et al., 2016). *Lactobacilli* constitute more than 50% of the total commensal ecology and maintain a pH of 3.8 and 4.5 with other bacterial species, which is considered normal (Miller et al., 2016). Together with their antibacterial properties and immunological factors, *Lactobacilli* form the first line of defense against dysbiosis and infections. To date, more than 120 *Lactobacillus* species have been recognized and dozens of them are populated in the vagina (Mendling, 2016). In this study, s_unclassified_g_Lactobacillus was the most dominant species whose proportion was higher in control compared with single HPV genotype and multiple HPV genotype infections. Our results suggested that the proportion of *Lactobacillus* and other bacterial spp. was associated with HPV infection, and the types of HPV genotype infection such as s_unclassified_g_Lactobacillus abundance were substantially lower in HPV-52, -58, and -16 compared with HPV-33, other HR-HPV, and LR-HPV.

The underlying molecular mechanisms of how vaginal microbiota might influence the HPV persistent infection are still unknown. *Lactobacillus* spp. can produce several antimicrobial agents, including lactic acid (Boskey et al., 2001), which protect against sexually transmitted and other infections (Alakomi et al., 2000; O'Hanlon et al., 2011; Motevaseli et al., 2013). Our results show a relatively low abundance of *Lactobacilli* in women with single HPV genotype or multiple HPV genotype infection, which is consistent with a previously reported study in which they found overall high HPV infectivity and an elevated risk of multiple HPV genotype infections. Women have higher pH levels due to lack of *Lactobacillus* spp. (Clarke et al., 2012). Further, a high inflammatory response could improve viral clearance, but the immune-modulating bacteria may play a role in the progression of diseases (Khan et al., 2012; Quevrain et al., 2016). In our study, *Proteobacteria*, s_unclassified_f_Rhizobiaceae, and s_sneathia_sanguinegens were only found in HPV-infected women, which requires further investigation to confirm their role in pathogenesis. Further investigations are needed to establish the role of bacteria in immunomodulation in HPV infection and its persistence.

Nowadays, HPV vaccines (Gardasil and Cervarix) have been approved in P.R. China and have become a hope for the possible elimination of HPV-related diseases (Brotherton, 2017; Huh et al., 2017). Despite these exciting scientific breakthroughs, many low-resource populations will not benefit from the vaccines due to technological, economic, and religious barriers. Consequently, it is important to identify alternative, evidence-based, and cost-effective intervention strategies that can be implemented to reduce HPV infection. Improving understanding of the vaginal microbiota offers opportunities to maximize a woman’s first line of defense and contribute to developing practical and low-cost interventions to reduce her susceptibility to HPV infection. In a pilot study of 54 HPV-positive women diagnosed with low-grade squamous intraepithelial lesions, their daily consumption of a probiotic drink containing *Lactobacillus casei* appeared helpful in the clearance of HPV infection (Verhoeven et al., 2013). Therefore, we hypothesize that a personalized selection of probiotics would be most effective for women’s health and protection. It will be interesting and valuable to determine whether probiotic intervention can effectively reduce productive viral infection and lead HPV to a clinically “latent” situation in future studies.

The strength of this study includes the comparison of microbial composition and diversity of vaginal microbiota between single infections, multiple infections, high-risk infections, and low-risk infections, as well as different combinations of HPV genotypes such as HR–HR, HR–LR, and LR–LR. The weaknesses of our study include a small sample size, lack of HPV burden data, and other potential risk factors such as contraceptive methods, which would be critical for a better understanding of the association between HPV and vaginal microbiota.

## Conclusions

Our results suggest that HPV infection types, i.e., single HPV, HR-HPV, and multiple HPV genotype infections, influence the composition of the vaginal microbiota, particularly non-*Lactobacillus*. Further studies on HPV genotype level infection and vaginal microbiota diversity are necessary to uncover more mysteries of their association and provide a promising therapeutic target as well as low-cost future therapeutic strategies.

## Data availability statement

The datasets generated for this study were submitted to the NCBI under the accession number (PRJNA799456).

## Ethics statement

The studies involving human participants were reviewed and approved by Ethics Committee at Kunming University of Science and Technology. The patients/participants provided their written informed consent to participate in this study.

## Author contributions

XX and ZB conceived and designed the research plan and supervised the study. ZB, YS, YL and SL finished the lab work, bioinformatics process, and statistical analysis. XW and YL performed sample collection. XX and ZB were responsible for manuscript writing and revising. All authors contributed to the article and approved the submitted version.
